# Interactions between hematological biomarkers of virus infection and immune cells in mediating distant metastasis in nasopharyngeal carcinoma: insights into prognosis and induction chemotherapy administration

**DOI:** 10.2478/raon-2026-0005

**Published:** 2026-02-06

**Authors:** Shuqi Li, Biyun Chen, Di Cao, Chao Luo, Zhiying Liang, Ian Kou, Ge Ren, Wenjie Huang, Guangying Ruan, Lizhi Liu, Haojiang Li, Siyu Zhu, Ai Fei

**Affiliations:** Department of Radiology, Sun Yat-sen University Cancer Center, State Key Laboratory of Oncology in South China, Collaborative Innovation Center for Cancer Medicine, Guangdong Key Laboratory of Nasopharyngeal Carcinoma Diagnosis and Therapy, Guangdong Provincial Clinical Research Center for Cancer, Guangzhou, Guangdong, China; Department of Mathematics, Faculty of Science and Technology, University of Macau, Macau China; Department of Health Technology and Informatics, The Hong Kong Polytechnic University, Hung Hom, Kowloon, Hong Kong, China

**Keywords:** nasopharyngeal carcinoma, Epstein-Barr virus, hepatitis B, prognosis, induction chemotherapy

## Abstract

**Background:**

Considering the increased metastatic risk in hepatitis B surface antigen (HBsAg)-positive patients with nasopharyngeal carcinoma (NPC), we aimed to investigate the interactions among HBsAg, tumor burden indicators, and immune function in the accurate stratification of prognosis and treatment for this specific cohort.

**Patients and methods:**

We retrospectively analysed 1650 pathologically-confirmed patients with NPC from two centers and performed interaction and mediation analyses among HBsAg, plasma Epstein-Barr virus (EBV) DNA load, and absolute lymphocyte count (ALC), concerning distant metastasis. A 1:1 random matched-paired analysis was performed to evaluate survival according to risk and treatment stratification. Treatment-related adverse events were also compared.

**Results:**

Overall, 17.3% (285/1650) of patients tested positive for HBsAg. Significant interactions occurred between HBsAg and low ALC (≤ 1.9×10^9^/L) (HL), as well as between HBsAg and high plasma EBV DNA load (> 4000 copies/mL) (HE), both independently predicting poor distant metastasis-free survival (DMFS). The influence of T and N staging on tumor metastasis was mediated by HL (+) and HE (+), respectively. Among patients with stage III–IVa NPC, interaction associations presented with a worse 5-year DMFS and higher rates of severe neutropenia and leukopenia among those treated with additional induction chemotherapy (IC) than among those treated with radiochemotherapy alone.

**Conclusions:**

Interactions exist between HBsAg positivity and high EBV/low ALC, mediating the effects of tumor staging and distant metastasis. The collective influence of viral infection, tumor burden, and reduced immune cells leads to worse DMFS in patients with HBsAg-positive NPC, requiring a tailored treatment beyond IC.

## Introduction

Despite significant advances in survival improvement obtained by multi-modal treatment strategies^1–3^, distant metastasis still accounts for the highest percentage (11.9%) of all identified failure types in nasopharyngeal carcinoma (NPC).^4^ The plasma Epstein-Barr virus (EBV) DNA level in NPC correlates with tumor burden and treatment response^5,6^, independently predicting distant metastasis.^7–9^ Elevated EBV DNA load increases susceptibility to distant metastasis.^10,11^ Moreover, we previously demonstrated that the synergistic effect between plasma EBV DNA load and hepatitis B surface antigen (HBsAg) contributes to the development of metastasis in NPC.^12^ Although the role of HBsAg in distant metastasis is debatable^13,14^, Weng *et al*.^15^ reported a 3.7-fold higher metastatic risk in patients with early-stage NPC with HBsAg positivity. Further research is needed to address the potential confounders that influence the impact of HBsAg on NPC outcomes.

According to the National Comprehensive Cancer Network (NCCN) and American Society of Clinical Oncology guidelines^16,17^, induction chemotherapy (IC) combined with concurrent chemoradiotherapy (CCRT) is the recommended standard treatment for patients with NPC at high risk of metastasis. Patients with NPC infected with hepatitis B virus (HBV) tend to receive IC owing to their increased risk of metastasis. However, previous studies suggest that patients with HBV infection may not benefit from additional IC and may even present with a worse prognosis.^18,19^ One possible explanation is that chronic HBV infection can impair the adaptive response, resulting in functional T-cell inhibition and tumor immune evasion.^20,21^ Moreover, potential immunosuppression because of chemotherapy could cause further liver damage and HBV reactivation, undermining the therapeutic benefit of IC resulting in a negative prognostic effect.^22^

The absolute lymphocyte count (ALC) in peripheral blood is a valuable indicator of the host’s immune function and can be used to predict the prognosis of various diseases.^23,24^ A low ALC usually suggests a weak immune response against tumors, potentially leading to disease progression and poor outcomes for patients with NPC.^25,26^ Furthermore, a high pre-treatment ALC predicts a favourable prognosis, indicating the benefits of adding cisplatin chemotherapy to radiotherapy in oropharyngeal cancer.^27^ Nevertheless, the association between HBsAg status, EBV DNA load, ALC, and their collaborative influence on survival outcomes and chemotherapy response in patients with NPC remains unclear and requires further in-depth exploration.

Therefore, the principal aim of our study was to explore the complex interplay between HBsAg, EBV, and pre-treatment ALC and their correlation with distant metastasis in NPC. We sought to determine whether these factors could serve as predictive markers for the benefits derived from the addition of IC in a large, homogeneous cohort of patients with NPC from two independent hospitals.

## Patients and methods

### Patient enrolment

**Figure 1.**
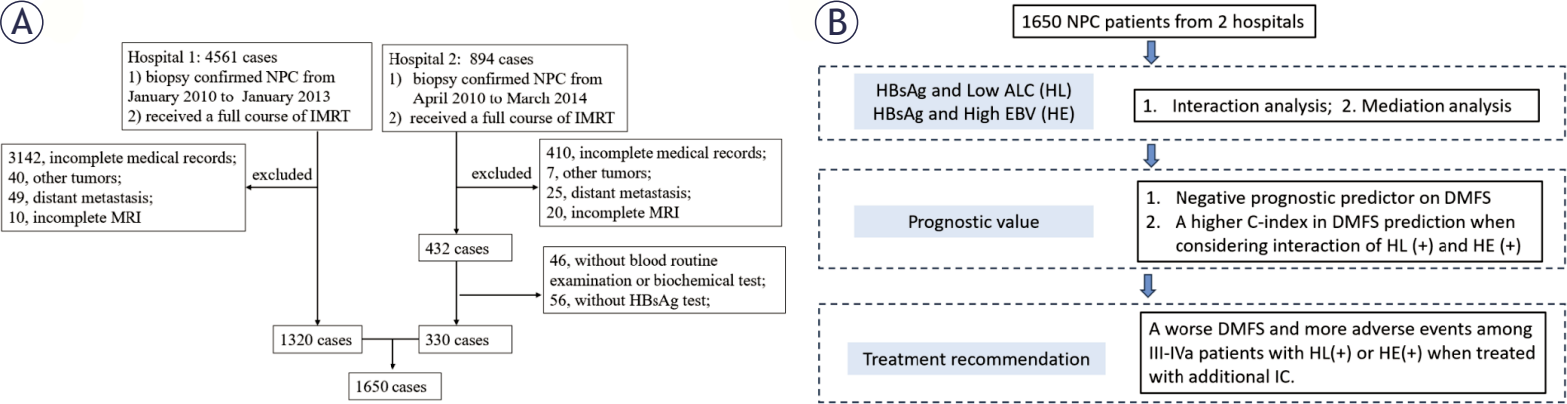
Patient enrolment and study flowchart. **(A)** Patient enrolment flowchart from the two hospitals. **(B)** Statistical analysis flowchart. **Note 1**. The time involved in the exclusion criteria is set at the time before treatment. As all enrolled patients in Hospital 1 had data from routine blood examinations, biochemical tests, and HBsAg tests, no other patients were further excluded from Hospital 1. **Note 2**. Complete MRI included scanning of the nasopharynx and neck regions, which can be used for restaging assessment of the 8th TNM staging system. **Note 3**. Low ALC indicated an absolute lymphocyte count ≤ 1.9 ×10^9^/L. High EBV indicated plasma EBV DNA level > 4 ×10^3^ copies/mL. ALC = absolute lymphocyte count; HBsAg = hepatitis B surface antigen; HE = interaction between HBsAg (+) and high EBV; HL = interaction between HBsAg (+) and low ALC; IC = induction chemotherapy; MRI = magnetic resonance imaging; OS = overall survival; non_IC = treated without induction chemotherapy; + = positive; - = negative

A retrospective evaluation was conducted on a total of 1650 patients with pathologically confirmed NPC at two centres between January 2010 and March 2014 (Figure 1A). The inclusion criteria were a pathological diagnosis of NPC and complete treatment with intensity-modulated radiotherapy (IMRT). The exclusion criteria were as follows: (i) incomplete medical history and hematological test (plasma EBV DNA, blood cell count, blood biochemistry, HBV, and liver function) record, (ii) concurrent cancer, (iii) distant metastasis upon initial presentation, and (iv) incomplete MRI for restaging.

Patients underwent quarterly evaluations during the initial 2 years and biannual evaluations in the subsequent 3 years. The principal endpoint of this study was distant metastasis-free survival (DMFS), which was calculated from treatment initiation to the occurrence of distant metastasis. Overall survival (OS) was regarded as the secondary endpoint and was calculated from treatment initiation to the date of death.

### Measurement of hematological markers

Pre-treatment peripheral blood samples were obtained to measure hematological markers. Plasma EBV DNA load was determined using real-time fluorescence quantitative polymerase chain reaction and classified as a dichotomous variable with a cut-off value of 4×10^3^ copies/mL.^28^ The complete blood count from the pre-treatment routine blood test was examined using a Sysmex XN-9000 automated hematology analyser (Sysmex, Kobe, Japan). The cut-off value for the complete blood cell count was divided by the median value. Inflammatory indicators, including lactate dehydrogenase (LDH) level, prognostic nutritional index (PNI, PNI = serum albumin [g/L] + 5 × ALC [×10^9^/L])^29^, and absolute lymphocyte and leukocyte counts, were included in the analysis to assess their impact on DMFS. Enzyme-linked immunosorbent assay was used to detect HBsAg status, with levels > 0.05 IU/mL considered HBsAg positive. Alanine transaminase (ALT) and aspartate aminotransferase (AST) levels were recorded to evaluate liver function, with normal ranges of 9–50 and 15–40 U/L, respectively.

### Treatment

The treatment strategy and recording principle of treatment-related acute adverse events are provided in Supplementary materials.

### Statistical analyses

Figure 1B shows the statistical analysis procedure. Regarding patient characteristics, the Student’s *t*-test and Chi-square or Fisher’s exact test were used to compare continuous and categorical variables, respectively. Association rules^12^ using “arules” and “arulesViz” were used to explore the association between HBsAg status and inflammatory blood indicators in distant metastasis. Univariate analysis with a log-rank test was used to select confounding factors related to DMFS among patients with NPC. Interaction associations were explored between HBsAg and inflammatory blood indicators as well as between HBsAg and plasma EBV DNA load according to the interaction formula: *Z = aX + bY + c(XY)*.

Logistic regression analysis was performed to assess the associations between variables. Mediation analysis was performed to investigate the mediating effect of tumor staging (exposure) on distant metastasis (outcome) by the hematological indicator using the “mediation” package. Bootstrapping with 1000 bootstrap samples was used to estimate the mediation proportion. A directed acyclic graph (DAG) was plotted to show the associations between tumor staging, interaction items, and tumor-distant metastases.

A 1:1 random matched-paired analysis was conducted within the total cohort to assess the prognostic value and IC-related survival using prognostic variables including T and N stage, plasma EBV DNA load, and age group. Multivariate Cox regression analysis using Kaplan–Meier plots was performed to calculate hazard ratios (HRs) with 95% confidence intervals (CIs). The total cohort was divided into training and testing cohorts at a 1:1 ratio for model establishment. Harrel’s concordance index (C-index) was calculated to assess the DMFS prediction ability of the various models. Pairwise comparisons of the C-indices were performed using U-statistics.

All statistical analyses were conducted using R packages such as stats, arules, survival, arulesViz, mediation, Hmisc, rms, maxstat, ggplot2, apriori, mediation, and survminer (https://www.r-project.org/). A two-tailed *P* < 0.05 was considered statistically significant.

## Results

### Basic characteristics and univariate analysis

The median (interquartile range) age of the 1650 patients with NPC from the two institutions was 46 (38–54) years, with 1221 (74.0%) of them being male. Overall, 85 (17.3%) patients were HBsAg positive. The median (interquartile range) follow-up time was 61.80 (45.93–70.06) months. In addition, 209 patients developed distant metastases at the end of the follow-up.

There were no differences in tumor burden, including tumor staging and plasma EBV DNA load, between the HBsAg (+) and HBsAg (-) groups. Both groups experienced comparable incidence rates of distant metastases (13.3% *vs*. 12.5%, *P* = 0.771). The age at cancer diagnosis in the HBsAg (+) group was lower than that in the HBsAg (-) group. Pretreatment ALT and AST levels were higher in the HBsAg (+) than in the HBsAg (-) group. The median value for ALC was 1.9×10^9^/L, which was consistent in both hospitals. Therefore, ALC was categorized as low (≤ 1.9) or high (> 1.9) in the subsequent analysis (Table 1).

**Table 1.** Basic patient characteristics and univariate analysis

Variables	Total	HBsAg (-)	HBsAg (+)	Chi-square	DMFS	
	(n = 1650)	(n = 1365)	(n = 285)	*P* value	5-year	Log-Rank *P*
Age group				< 0.001		0.548
≤ 45	821 (49.8%)	651 (47.7%)	170 (59.6%)		87.06	
> 45	829 (50.2%)	714 (52.3%)	115 (40.4%)		85.87	
Sex				0.035		0.424
Male	1221 (74.0%)	996 (73.0%)	225 (78.9%)		86.05	
Female	429 (26.0%)	369 (27.0%)	60 (21.1%)		87.64	
Plasma EBV DNA load (copies/ml)			0.536			< 0.001
≤ 4×10^3^	1071 (64.9%)	891 (65.3%)	180 (63.2%)		90.49	
> 4×10^3^	579 (35.1%)	474 (34.7%)	105 (36.8%)		78.83	
Histologic type^a^				0.391		0.463
WHO type 1/2	63 (3.8%)	55 (4.0%)	8 (2.8%)		82.71	
WHO type 3	1587 (96.2%)	1310 (96.0%)	277 (97.2%)		86.63	
T stage^b^				0.186		< 0.001
T1	415 (25.2%)	350 (25.6%)	65 (22.8%)		93.54	
T2	201 (12.2%)	172 (12.6%)	29 (10.2%)		85.97	
T3	649 (39.3%)	521 (38.2%)	128 (44.9%)		86.49	
T4	385 (23.3%)	322 (23.6%)	63 (22.1%)		78.71	
N stage^b^				0.962		< 0.001
N0	331 (20.1%)	272 (19.9%)	59 (20.7%)		95.22	
N1	934 (56.6%)	774 (56.7%)	160 (56.1%)		87.63	
N2	277 (16.8%)	231 (16.9%)	46 (16.1%)		79.05	
N3	108 (6.5%)	88 (6.4%)	20 (7.0%)		66.79	
Stage^b^				0.590		< 0.001
I	139 (8.4%)	119 (8.7%)	20 (7.0%)		97.55	
II	361 (21.9%)	302 (22.1%)	59 (20.7%)		93.05	
III	677 (41%)	551 (40.4%)	126 (44.2%)		86.81	
IVa	473 (28.7%)	393 (28.8%)	80 (28.1%)		77.13	
IC treatment				0.188		< 0.001
non_IC	809 (49.0%)	659 (48.3%)	150 (52.6%)		90.10	
IC	841 (51.0%)	706 (51.7%)	135 (47.4%)		82.91	
HBsAg status				< 0.001		0.612
HBsAg (-)	1365 (82.7%)	1365 (100.0%)	0		86.60	
HBsAg (+)	285 (17.3%)	0	285 (100.0%)		85.87	
ALC^c^ (10^9^/L)	1650 (100%)	1.9 (1.5–2.3)	1.9 (1.5–2.4)	0.514	-	0.663
ALC group (10^9^/L)				0.694		0.223
> 1.9	742 (45.0%)	617 (45.2%)	125 (43.9%)	160 (56.1%)	87.58	
≤ 1.9	908 (55.0%)	748 (54.8%)			85.55	
ALT^c^ (U/L)	1650 (100.0%)	19.9 (16–24.9)	24 (18.9–34)	< 0.001	-	0.256
AST^c^ (U/L)	1650 (100.0%)	20 (16.2–26)	24.2 (20–36.5)	< 0.001	-	0.273

a According to the 2005 World Health Organization classification of tumors;

b According to the 8th edition of the AJCC staging system;

c continuous variable

1 ALC = absolute lymphocyte count; ALT = alanine aminotransferase; AST = aspartate transaminase; DMFS = distant-metastasis-free survival; EBV = Epstein-Barr virus; HBsAg = hepatitis B surface antigen; IC = induction chemotherapy

In the univariate analysis, the pre-treatment plasma EBV DNA load, tumor staging, and treatment strategy were related to DMFS. Notably, HBsAg was not an independent confounder for DMFS. Similarly, whether treated as a continuous or categorical variable, ALC was not an independent prognostic predictor. Considering the pretreatment prediction, plasma EBV DNA load and tumor staging (T and N stages) were selected as the final confounding factors in subsequent analyses.

### Association and interaction between HBsAg and hematological indicators on distant-metastasis-free survival

**Figure 2.**
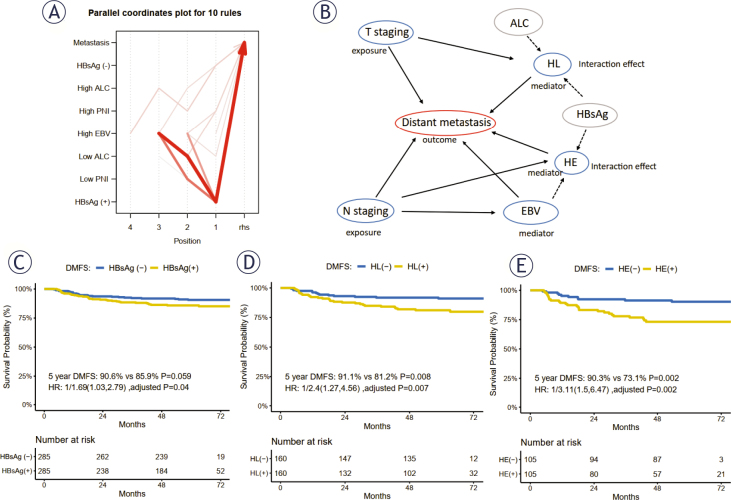
Association rules, directed acyclic graph, and prognostic value of HBsAg, EBV, and ALC on DMFS. **(A)** The top 10 association rules related to HBsAg (+) demonstrated strong associations among high EBV, low ALC, and HBsAg (+) with distant metastasis in patients with NPC. **(B)** The directed acyclic graph (DAG) shows the associations among variables. Significant interactions were observed between HBsAg and plasma EBV DNA load, HBsAg, and ALC, defined as HE and HL. In the mediation analysis, EBV and HE mediated the effect of N staging on distant metastasis, and HL mediated the effect of T staging on distant metastasis. After 1:1 random matched analysis **(C–E)**, HBsAg (+), HL (+), and HE (+) were the independent adverse prognosticators of DMFS in patients with NPC. **Note 1**. The width of the curves represents the occurrence frequency of each association rule. X-axis: the number of the position indicates the number of variables involved in the rule. Rhs was set as distant metastasis. The plasma EBV DNA load classification is divided by the cut-off of 4 ×10^3^ copies/mL. The classification of ALC and PNI is divided by the cut-off with its median value. The original rules are presented in Supplementary Table 1. **Note 2**. Supplementary Tables 2 and 4 show the interaction and mediation analyses, respectively. **Note 3**. The 1:1 random matched survival analyses were performed with the match factors of T and N stages, plasma EBV DNA, and age group. The *P* values were calculated using a log-rank test. ALC = absolute lymphocyte count; DMFS = distant metastasis-free survival; EBV = pre-treatment plasma EBV DNA level; HBsAg = hepatitis B surface antigen; HE = interaction between HBsAg (+) and high EBV; HL = interaction between HBsAg (+) and low ALC; lhs = left-hand side; PNI = prognostic nutritional index; rhs = right-hand side; WBC = white blood cell; +, positive; -, negative

Overall, 1379 association rules were identified among the status of T and N stages, HBsAg, plasma EBV DNA load, and immune-related hematological indicators on DMFS (including white blood cells, ALC, LDH, and PNI). The variables from the HBsAg positivity-related rules within the top 30 rules (Supplementary Table 1) were selected for further assessment. The top 10 rules concerning HBsAg (+) (Supplementary Table 1 and Figure 2A) demonstrated strong associations among HBsAg (+), high EBV, and low ALC with distant metastasis.

Significant interaction and association were found between HBsAg and low ALC on DMFS (HR = 2.54, 95% CI: 1.13–5.69, *P* = 0.023). Considering the confounding factors, independent associations were observed between HBsAg and low ALC (HL) (HR = 2.67, 95% CI: 1.19–5.99, *P* = 0.017) and between HBsAg and high plasma EBV DNA load (HE) (HR = 2.27, 95% CI: 1.08–4.81, *P* = 0.031), which were independent adverse prognostic predictors for DMFS in patients with NPC. Moreover, there was no interaction or association between low ALC and high plasma EBV DNA load (Supplementary Table 2).

HBsAg positivity did not independently predict DMFS, but demonstrated independence when factoring in the interaction effect of HL and HE on DMFS prediction (Table 2). Although the C-index of the proposed model incorporating tumor staging with interaction was similar to that of the TN+EBV model in predicting DMFS, it significantly improved in both training (0.712 *vs*. 0.688, *P* = 0.002) and testing (0.695 *vs*. 0.678, *P* = 0.047) cohorts compared with the TN model using T stage and N stage (Supplementary Table 3). The decision curve analysis (Supplementary Figure 1) demonstrated that the proposed model yielded higher positive net benefits in the total cohort than the TN and TN+EBV models, indicating a better discrimination ability for DMFS prediction when HL (+) and HE (+) were considered.

**Table 2.** Consideration of interaction effect of HL and HE in distant-metastasis-free survival (DMFS) prediction

Variables	DMFS		Variables	DMFS	
	HR (95% CI)	*P*		HR (95% CI)	*P*
Step 1: Without interaction item			Step 2: Interaction consideration		
NA			HBsAg (+) × Low ALC (HL)	2.56 (1.14–5.76)	0.023
NA			HBsAg (+) × High EBV (HE)	2.16 (1.02–4.58)	0.044
HBsAg (-/+)	1.12 (0.79–1.6)	0.524	HBsAg (-/+)	0.38 (0.17–0.88)	0.024
NA			ALC (≤ 1.9 *vs*. > 1.9)*	0.94 (0.69–1.27)	0.692
EBV (≤ 4 *vs* > 4)^#^	1.56 (1.16–2.08)	0.003	EBV (≤ 4 *vs*. > 4)^#^	1.34 (0.97–1.84)	0.075
T stage			T stage		
T1	1 (reference)		T1	1 (reference)	
T2	1.61 (0.94–2.76)	0.084	T2	1.66 (0.97–2.84)	0.066
T3	1.62 (1.04–2.52)	0.032	T3	1.62 (1.04–2.52)	0.034
T4	2.62 (1.67–4.1)	< 0.001	T4	2.63 (1.68,4.13)	< 0.001
N stage			N stage		
N0	1 (reference)		N0	1 (reference)	
N1	2.01 (1.18–3.43)	0.010	N1	2.01 (1.18–3.43)	0.010
N2	3.25 (1.83–5.8)	< 0.001	N2	3.34 (1.87–5.95)	< 0.001
N3	5.09 (2.69–9.61)	< 0.001	N3	5.28 (2.8–9.97)	< 0.001

# EBV (×1000 copies/mL),

* ALC (×10^9^/L); HR and *P* values were calculated using multivariate Cox regression analysis.

1 ALC = absolute lymphocyte count; CI = confidence interval; DMFS = distant metastasis-free survival; EBV = pre-treatment plasma EBV = Epstein-Barr virus DNA levels; HBsAg = hepatitis B surface antigen; HE = interaction between HBsAg (+) and high EBV; HL = interaction between HBsAg (+) and low ALC; HR = hazard ratio; NA = not applicable; + = positive; - = negative

### Mediation analysis of interaction items on the associations between tumor stage and distant metastasis

Logistic regression analysis showed that T stage was associated with HL (+), while N stage was associated with HE (+) and plasma EBV DNA load. In the mediation analysis, the HRs for exposure variables were adjusted for T and N stage and plasma EBV DNA load, according to the situation of the mediator. After adjusting for HL (+) and confounding factors, the HR for the advanced T stage was 1.56 (95% CI: 1.14–2.15). The mediation proportion of HL was 7.58% (95% CI: 0.54%–26.00%, *P* = 0.032) within the association between T stage and distant metastasis. The HR for advanced T stage was higher when not adjusted for HL (+). Moreover, EBV and HE (+) mediated the association between N stage and distant metastasis. The mediation of EBV accounted for 19.3% (95% CI: 7.28%–36.00%, *P* = 0.004) of N stage on distant metastasis, while that of HE accounted for 5.39% (95% CI: 0.37–14.0, *P* = 0.032). The HR for advanced N stage decreased when mediated by EBV (from 2.35 to 2.01). Conversely, the HR for advanced N stage increased when mediated by HE (from 2.01 to 2.05) (Table 3). The associations among tumor staging, interaction items, and distant metastasis are presented on the DAG (Figure 2B).

**Table 3.** The changes in the hazard ratio of tumor staging when HL and HE are considered as mediators in the multivariate Cox regression model on distant-metastasis-free survival (DMFS)

Mediator	Exposure	Unadjusted for mediator		Adjusted for mediator		Mediation proportion %	
		HR (95%CI)	*P*	HR (95% CI)	*P*	HR (95%CI)	*P*
HL (+)	T stage						
	T1-2	1 (Reference)	0.004	1 (Reference)	0.006		
	T3-4	1.59 (1.16-2.18)		1.56 (1.14-2.15)		7.58 (0.538-26)	0.032
EBV	N stage						
	N0-1	1 (Reference)	< 0.001	1 (Reference)	< 0.001		
	N1-2	2.35 (1.78-3.11)		2.01 (1.51-2.69)		19.3 (7.28-36)	0.004
HE (+)	N stage						
	N0-1	1 (Reference)	< 0.001	1 (Reference)	< 0.001		
	N1-2	2.01 (1.51-2.69)		2.05 (1.53-2.74)		5.39 (0.37-14.0)	0.032

1 The mediation associations were: T stage (exposure) →HL (+) (mediator) →distant metastasis (outcome); N stage (exposure) →EBV (mediator) →distant metastasis (outcome); N stage (exposure) →HL (+) (mediator) →distant metastasis (outcome). ‘Unadjusted for mediator’ indicates that the item of the mediator and its related variable were not involved in the multivariate analysis. ‘Adjusted for mediator’ indicates that the mediator item and its related variable were considered in the multivariate analysis. Mediation proportion (%) represents the proportion of the effect of the mediator on DMFS to the total effect of exposure on DMFS.

1 ALC = absolute lymphocyte count; DMFS = distant metastasis-free survival; EBV = pre-treatment plasma EBV DNA level; HBsAg = hepatitis B surface antigen; HE = interaction between HBsAg (+) and high EBV; HL = the interaction between HBsAg (+) and low ALC

### Hazard risk of distant metastasis in patients with NPC with interaction effects

The 1:1 random matched-paired analysis using prognostic variables including T and N stage, plasma EBV DNA load, and age group revealed that HBsAg positivity was an independent negative factor for 5-year DMFS after adjusting for confounding factors (90.6% *vs*. 85.9%, *P* = 0.059; HR: 1.69, 95% CI: 1.03–2.79, adjusted *P* = 0.04; Figure 2C). Although ALC was not an independent prognostic factor for 5-year DMFS among patients with NPC (Supplementary Figure 2A), HL (+), as an interaction factor, significantly worsened the 5-year DMFS compared to that in patients with HL (-) (91.1% *vs*. 81.2%, *P* = 0.008; HR: 2.4, 95% CI: 1.274.56, adjusted *P* = 0.007). Furthermore, the 5-year DMFS of patients with NPC with HE interaction was significantly worse than that of patients without HE (90.3% *vs*. 73.1%, *P* = 0.002; HR: 3.11, 95% CI: 1.5–6.47, adjusted *P* = 0.002; Figure 2 D, E). When patients with NPC with HL (+) or HE (+) were excluded, the 5-year DMFS of patients with HBsAg positivity was insignificantly higher than that of those without HBsAg positivity (89.0% *vs*. 95.9%, *P* = 0.101), indicating the adverse prognostic role of interaction items (Supplementary Figure 2B). These findings indicate that HL (+) and HE (+) interactions might reveal the underlying reasons for the poor prognosis of patients with NPC who test positive for HBsAg.

### Outcomes of induction chemotherapy administration among HBsAg-positive patients with nasopharyngeal carcinoma

**FIGURE 3.**
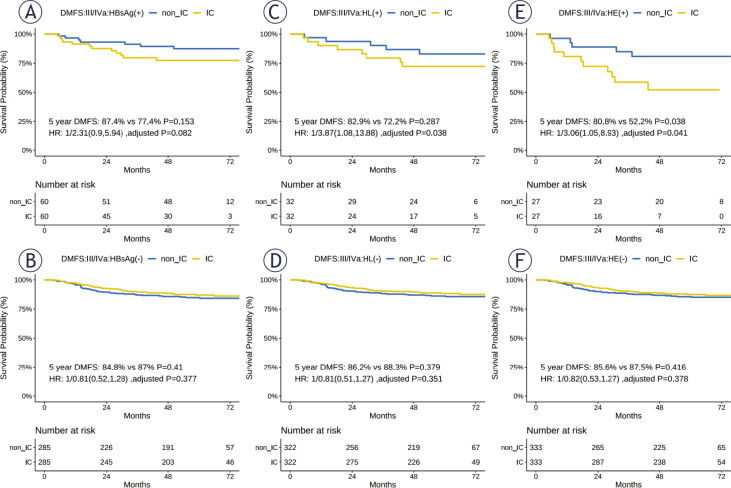
The 5-year DMFS among patients treated with and without induction chemotherapy. **Note 1**. The 1:1 random matched survival analysis was performed among stage III–IVa patients, with the match factors of T and N stages, plasma EBV DNA, and age group. The *P* values were calculated using a log-rank test. DMFS = distant metastasis-free survival; HBsAg = hepatitis B surface antigen; HE = interaction between HBsAg (+) and high EBV; HL = interactions between HBsAg (+) and low ALC; IC = induction chemotherapy; non_IC = treated without induction chemotherapy; + = positive; - = negative

Among patients with stage III–IVa NPC positive for HBsAg, the 5-year DMFS of the IC group was not significantly worse than that of the non-IC group. In patients with NPC with interaction associations, those who received additional IC presented with a worse 5-year DMFS than those who did not receive additional IC, both in HL (+) (82.9% *vs*. 72.2%, *P* = 0.287; HR: 3.87, 95% CI: 1.08–13.88, adjusted *P* = 0.038) and HE (+) (80.8% *vs*. 52.2%, *P* = 0.038; HR: 3.06, 95% CI: 1.05–8.93, adjusted *P* = 0.041) groups. Conversely, in patients without HBV infection and interaction association, there was an overlap in the 5-year DMFS, regardless of additional IC administration (Figure 3). Moreover, a better 5-year OS was observed among patients with stage III-IVa NPC receiving additional IC than among those treated without IC, whether they were classified into the HBsAg (-), HL (-), or HE (-) group. No significant treatment benefits were obtained in the 5-year OS for those in the HBsAg (+), HL (+), or HE (+) groups (Supplementary Figure 3).

The incidence rates of grade 3–4 immune-related hematological indicator reactions (including neutropenia and leukopenia) in HL (+) or HE (+) patients treated with IC were higher than those in patients treated without IC (Supplementary Figure 4). Moreover, the incidence rates of anemia, vomiting, nausea, and diarrhea were higher in HL (+) and HE (+) patients treated with IC than in those treated without IC (Supplementary Table 4). Therefore, additional IC worsened the prognosis of patients with NPC with HL or HE interaction associations and compromised their quality of life.

## Discussion

Our study primarily explored the complex interplay between HBsAg, EBV, and pre-treatment ALC and their correlation with distant metastasis in NPC. Regarding HBsAg positivity, we observed significant HL (< 1.9×10^9^/L) and HE (> 4000 copies/mL) interactions, emerging as negative predictors of DMFS in patients with NPC. The interaction variables (HL and HE) and plasma EBV DNA load played a mediating role in establishing a link between tumor staging and distant metastasis. Furthermore, patients with NPC experiencing the combined interaction effect of HL and HE exhibited a worse 5-year DMFS when subjected to additional IC treatment. The incidence rates of treatment-related grade 3–4 neutropenia and leukopenia were higher in patients with NPC with interaction effects when treated with additional IC than in those without additional IC treatment. Consequently, IC is not recommended for patients with NPC with HL or HE interactions.

The incidence rate of HBsAg positivity was 17.3% (285/1650), which was slightly higher than the 10–12% prevalence of HBV in southern China^30^, but within the expected range for NPC, with a reported rate of 24.8%.^22^ HBV infection increases the incidence of liver metastasis in patients with NPC^13^, implying a correlation between HBV infection and distant metastasis. However, it has been suggested that HBsAg does not independently impact the prognosis of NPC. We speculate that this effect might be influenced by potential confounding factors and interrelated variables, although the exact details remain unknown. In this regard, our largescale study demonstrated the interaction between HBsAg and high plasma EBV DNA load as an unfavorable prognostic factor for DMFS, consistent with our previous findings.^12^ Moreover, we evaluated the hematological immune-related indicators, which might reflect physical condition. Prior studies have suggested a correlation between ALC and NPC prognosis^25,26^; however, a conclusive relationship was yet to be established. Our results revealed that a decreased ALC induced an interaction effect on DMFS, indicating that impaired immunity contributes to distant metastasis in patients with NPC with HBsAg (+). Overall, our study revealed that HL and HE are independent prognostic factors for distant metastasis in NPC, shedding light on the synergistic and mediating effects of viral infections (HBV/EBV) and immune function (ALC) on distant metastasis in NPC. Our study offers a deeper insight into the potential pathways through which hepatitis B promotes NPC metastasis.

In the presence of hepatitis B, patients with high EBV and low ALC levels exhibited significantly lower DMFS rates. This phenomenon may initially be attributed to the impact of tumor burden^12^, followed by the important role of immunological disruption in HBV infection. Low ALC may lead to immune dysregulation in the context of HBV infection, thereby promoting distant metastasis. Moreover, studies have suggested that T-lymphocyte dysfunction is linked to HBV replication^31^, leading to a deficiency in cells that are crucial for immune responses against viruses and tumor infiltration. Patients with NPC often show changes in T cell subpopulations, particularly an imbalance in CD4+ and CD8+ T cells in the peripheral blood. Additionally, the expression of inhibitory receptors such as PD-1 and CTLA-4 on T cells in the peripheral circulation of patients with NPC leads to T cell exhaustion. Given the critical roles of T cell function in tumor development and immunity, we speculated that this may be the mechanism underlying the synergistic action observed in patients with NPC with HBsAg-positivity. However, the specific mechanism requires confirmation through cellular experiments and animal studies.

IC is currently recommended as a 2A treatment in the guidelines for locally advanced NPC.^17^ However, owing to its potential for increased toxicity, identifying suitable patients for IC is crucial for minimizing side effects and achieving precision treatment. Currently, no specific treatment recommendations are available for dual infection with HBV and EBV, because the relationship between HBV infection and NPC is unclear. However, the introduction of the interaction and mediation effects of HL and HE refines the prognostic impact of HBsAg in patients with NPC, facilitating more precise risk stratification for these patients. To our knowledge, this is the first study to reveal worse DMFS in patients with a synergistic effect when treated with IC than in those not treated with IC, suggesting that these patients do not benefit from IC and face an elevated risk of metastasis. Simultaneously, we observed a significant increase in severe neutropenia and leukopenia, indicating aggravated damage to the immune system. Immune system disruption may contribute to tumor progression.^25,26^ Thus, a decline in these blood indicators makes patients prone to infections and other issues, affecting their overall condition.

Furthermore, patients with advanced-stage disease and without interaction effects who received additional IC had overlapping 5-year DMFS rates. However, the noticeable advantage in the 5-year OS underscores the recommendation for stratified IC administration. Improving OS has been commonly regarded as the long-term benefit of IC in NPC treatment.^32^ This is possible because OS benefits are typically observed in various IC treatments, whereas DMFS benefits are limited to specific IC drugs (taxane- and gemcitabine-based IC regimens).^33^ Due to sample size limitations, we did not categorize the groups based on drug regimens. Therefore, the significant difference in OS but not DMFS may stem from the choice of IC drugs.

A previous study among patients with oropharyngeal squamous cell carcinoma reported that a higher pre-treatment ALC correlated with benefits from chemotherapy^27^, raising concerns about the ALC status in treatment management. Hence, caution is warranted when administering IC to patients with NPC who test positive for HBsAg, especially those with a low ALC. Conversely, elevating ALC levels may promote therapeutic benefits, underscoring the importance of personalized treatment in clinical trials. Consequently, monitoring plasma EBV DNA and ALC levels is essential for individuals with hepatitis B-associated NPC.

This study has some limitations. First, this was a retrospective study with limited data, highlighting the need for further confirmation using other databases or prospective studies. Second, the ALC indicator does not fully reflect the body’s immune status. Therefore, additional sequencing, cellular experiments, and animal studies are necessary to elucidate the immune mechanisms underlying the synergistic effects of HBV and EBV infection.

## Conclusions

Our study identified interactions between HBsAg positivity and low ALC, and between HBsAg positivity and high EBV levels. The mediation of the interaction items and plasma EBV DNA load was evident in connecting tumor staging with distant metastasis, revealing how HBsAg influences distant metastasis in NPC. The combined impact of viral infection, increased tumor burden, and diminished immune cell count contributed to poorer DMFS in patients with NPC. In HBsAg-positive patients with NPC, precise management beyond IC becomes crucial when they exhibit low ALC and elevated plasma EBV DNA levels. Hence, this study provides insights into the precise management of patients with NPC with HBV infection.

## Supplementary Material

Supplementary Material Details
