# Supplementary material for: Interactions between hematological biomarkers of virus infection and immune cells in mediating distant metastasis in nasopharyngeal carcinoma: insights into prognosis and induction chemotherapy administration
Source: Radiol Oncol. 2026 Feb 6;60(1):86–96. doi: 10.2478/raon-2026-0005 (PMC13012374; doi:10.2478/raon-2026-0005)
Supplement: Supplementary file 1 — Supplementary Material Details [file raon-2026-0005_sm.pdf]

Interactions between hematological biomarkers of virus infection and immune cells in mediating distant metastasis in nasopharyngeal carcinoma: insights into prognosis and induction chemotherapy administration

Shuqi Li, Biyun Chen, Di Cao, Chao Luo, Zhiying Liang, Ian Kou, Ge Ren, Wenjie Huang, Guangying Ruan; Lizhi Liu, Haojiang Li, Siyu Zhu, Ai Fei

doi: 10.2478/raon-2026-0005

Treatment

All patients underwent intensity-modulated radiotherapy following international guidelines for target delineation. Primary tumors were irradiated with a dose of 68 Gy, while lymph nodes received 60–64 Gy for planning target volume. The high-risk, low-risk, and cervical regional lymph node areas for the clinical target volume received 60, 54, and 54 Gy of radiation, respectively. Radiotherapy consisted of 30 sessions over 5–6 weeks. Concurrent cisplatin-based chemotherapy was administered to 87.8% of patients weekly at 30–40 mg/m^2 or 80–100 mg/m^2 every 2–3 cycles over three weeks. Overall, 51.0% of the enrolled patients received 2–3 cycles of induction chemotherapy within 21 days before radiotherapy, comprising either 80 mg/m^2 cisplatin combined with 1000 mg/m^2 5-fluorouracil, 75 mg/m^2 cisplatin combined with 75 mg/m^2 docetaxel, or 60 mg/m^2 cisplatin combined with 600 mg/m^2 5-fluorouracil and 60 mg/m^2 docetaxel.

Palliative approaches, such as radiotherapy, chemotherapy, or surgery were provided for patients with recurrence or progression. Based on data availability, acute adverse events for patients in hospital 1 were documented according to the Common Terminology Criteria for Adverse Events (CTCAE) Version 4.0.[1]

References

National Cancer Institute. Common Terminology Criteria for Adverse Events (CTCAE). Version 4.0. 2009.

Supplementary figures

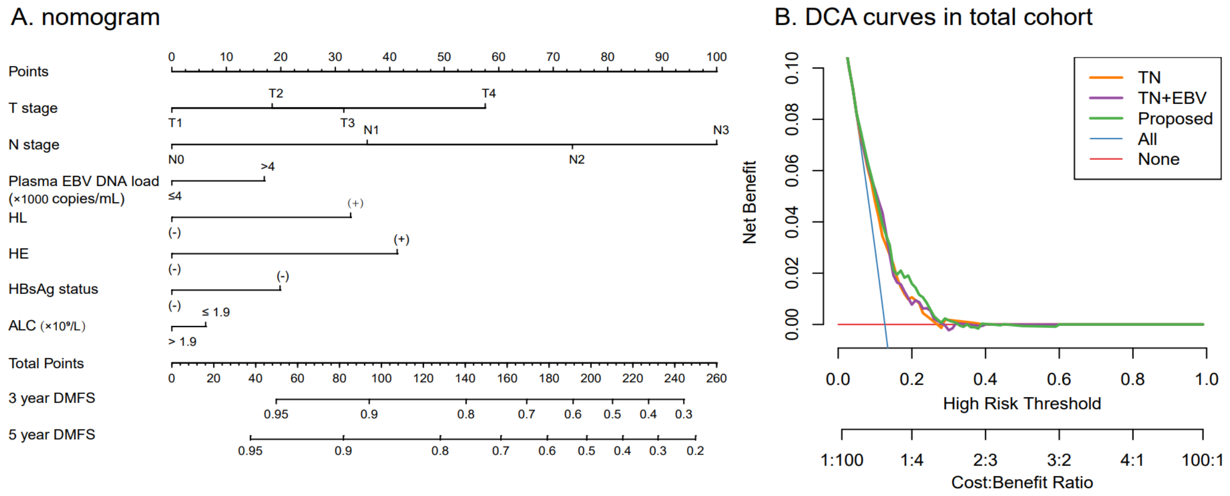

SUPPLEMENTARY FIGURE 1. Nomogram of the proposed model and decision curve analysis (DCA) of models in the total cohort. **(A)** Nomogram of the proposed model. **(B)** DCA curves of models in the total cohort.

The proposed model was established by incorporating HBsAg, HL, HE, ALC, and EBV status, as well as T and N stages. The TN+EBV model was established considering EBV status and T and N stages. The TN model was established using the T and N stages.

ALC = absolute lymphocyte count; DMFS = distant metastasis-free survival; HBsAg = hepatitis B surface antigen; HE = interaction between HBsAg (+) and high EBV; HL = interaction between HBsAg (+) and low ALC;. + = positive; - = negative

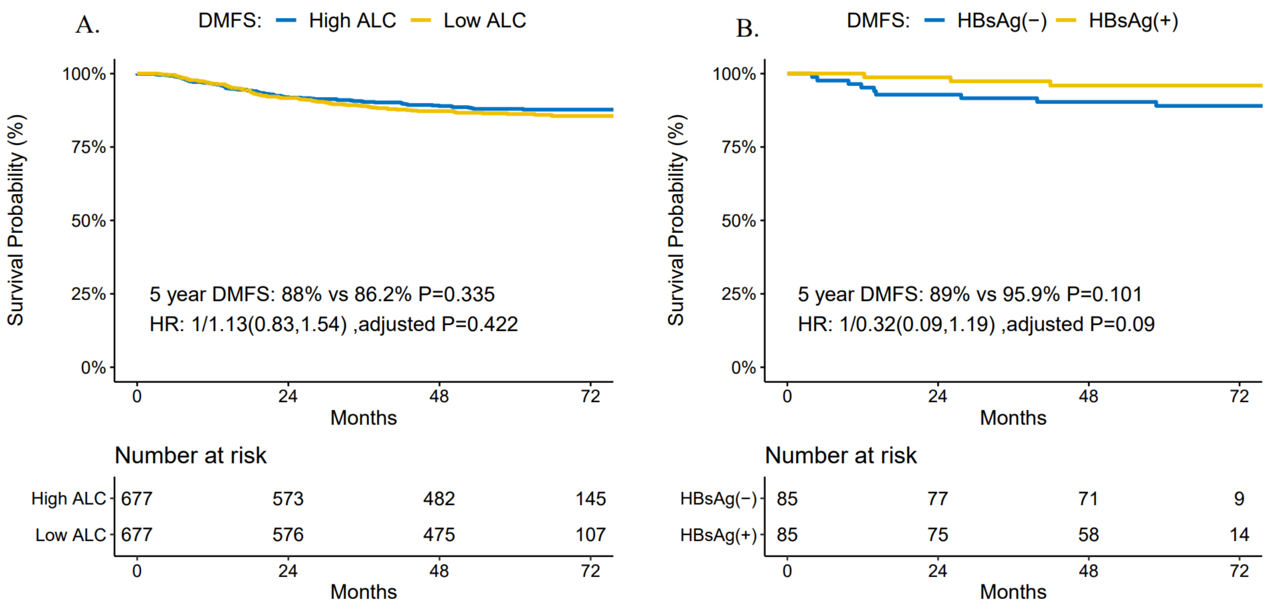

SUPPLEMENTARY FIGURE 2. Prognostic value of ALC and HBsAg in patients excluded interaction item. **(A)** ALC was divided into high and low ALC based on its median value of 1.9×10⁹/L. The 5-year DMFS rates among the high and low ALC patients overlapped. **(B)** Among patients without any interaction effect of HL and HE, the 5-year DMFS of patients with HBsAg (+) was not significantly higher than those with HBsAg (-).

ALC = absolute lymphocyte count; DMFS = distant metastasis-free survival; HBsAg = hepatitis B surface antigen; HE = interaction between HBsAg (+) and high EBV; HL = interaction between HBsAg (+) and low ALC; + = positive; - = negative

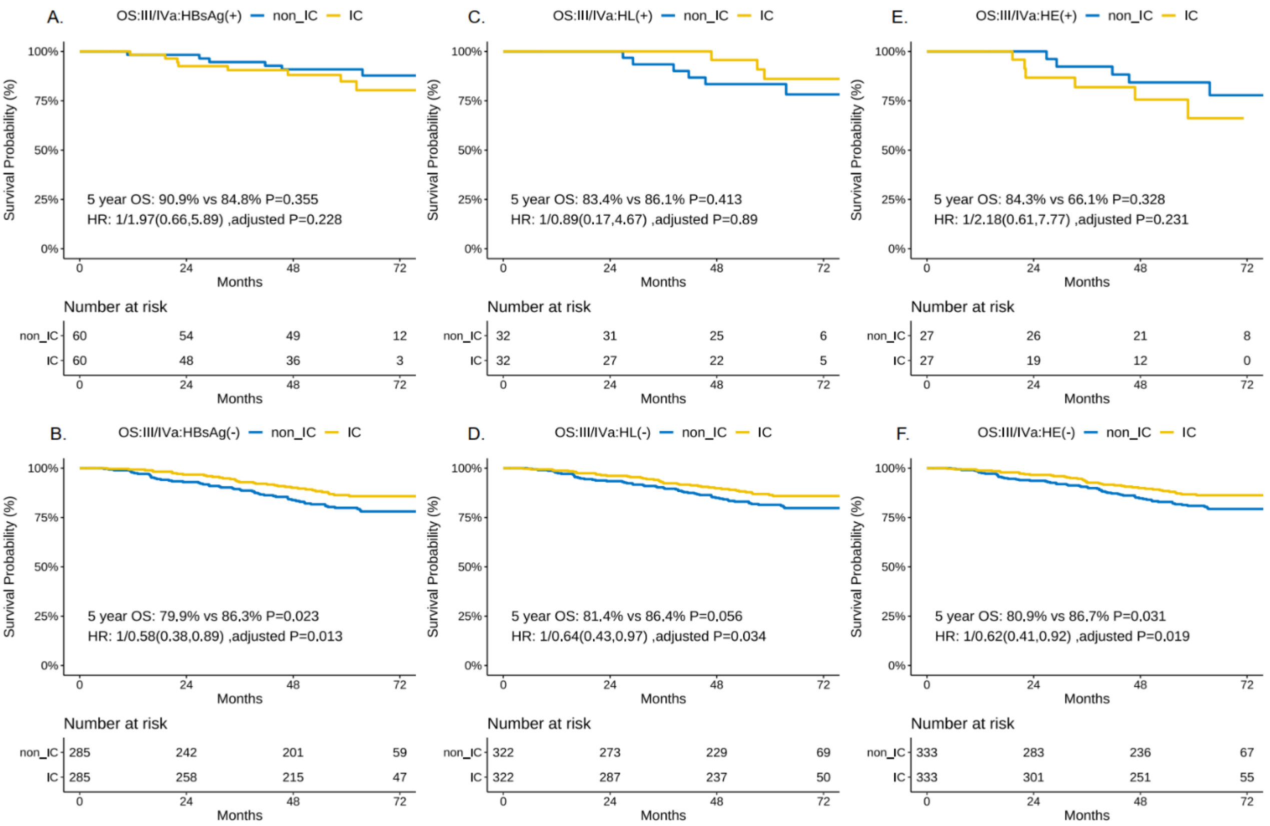

SUPPLEMENTARY FIGURE S3. The 5-year overall survival among patients treated with and without induction chemotherapy.

The 1:1 random matched survival analysis was performed among patients with stage III–IVa, with the match factors of T stage, N stage, plasma EBV DNA classification, and age. The *P* values were calculated using the log-rank test.

HBsAg = hepatitis B surface antigen; HE = interaction between HBsAg (+) and high EBV; HL = interaction between HBsAg (+) and low ALC; IC = induction chemotherapy; OS = overall survival; non_IC = treated without induction chemotherapy; + = positive; - = negative;

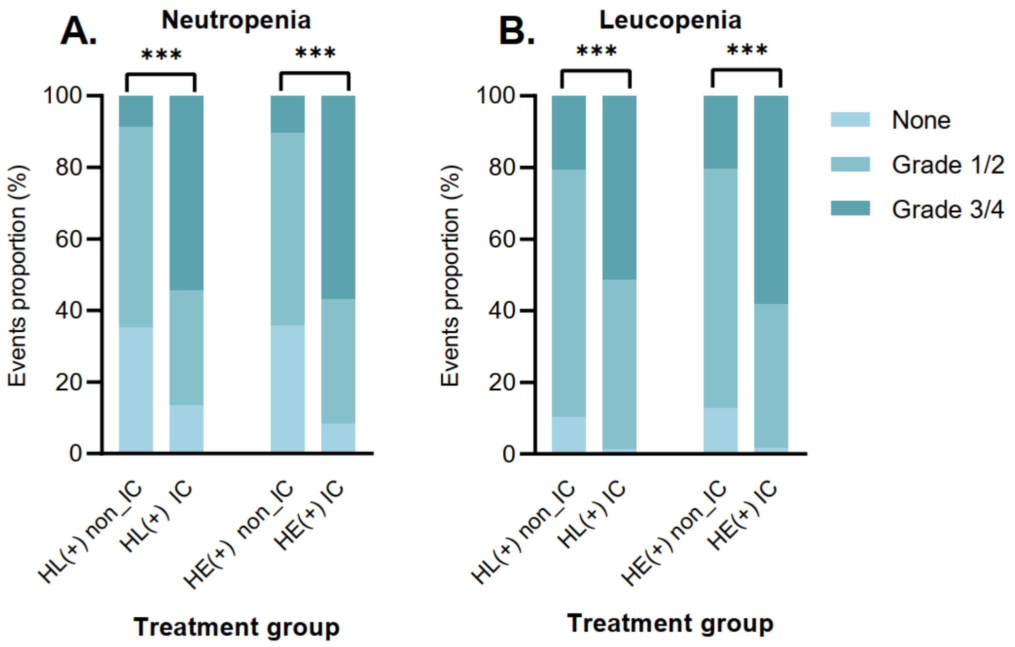

SUPPLEMENTARY FIGURE 4. Clustered column chart for neutropenia and leukopenia. Among patients with HL (+) and HE (+), the incidence rate of those who experienced grade 3/4 neutropenia and leukopenia was significantly higher in patients treated with IC than in those treated without IC.

Detailed statistics are presented in Supplementary Table 4.

ALC = absolute lymphocyte count; HE = interaction effect of HbsAg and high EBV; HL = interaction effect of HBsAg and low ALC; IC = induction chemotherapy; non_IC = those without induction chemotherapy; *** p < .001

Supplementary tables

SUPPLEMENTARY TABLE 1. Association rules

No.	LHS (Rules)	Support	Confidence	Coverage	Lift	Count
	**Top 30 rules associated with DMFS**					
1	{T4, N2, High LDH, High PNI}	0.007	0.524	0.013	4.135	11
2	{T4, N2, High WBC, High PNI}	0.007	0.462	0.016	3.644	12
3	{T4, N2, HBsAg (-), High PNI}	0.008	0.452	0.019	3.565	14
4	{T4, N2, High PNI}	0.009	0.441	0.021	3.483	15
5	{N2, HBsAg (-), Low ALC, High PNI}	0.007	0.407	0.016	3.216	11
6	{N3, HBsAg (-), High WBC, High PNI}	0.007	0.379	0.018	2.995	11
7	{N3, HBsAg (-), High LDH, High WBC}	0.007	0.364	0.020	2.871	12
8	{T3, HBsAg (+), High EBV, Low ALC}	0.008	0.361	0.022	2.851	13
9	{N3, High EBV, Low ALC, Low PNI}	0.008	0.359	0.024	2.834	14
10	{N2, Low ALC, High PNI}	0.007	0.353	0.021	2.786	12
11	{N3, High LDH, Low ALC, Low PNI}	0.007	0.353	0.021	2.786	12
12	{N3, High LDH, Low PNI}	0.008	0.350	0.024	2.763	14
13	{N3, Low WBC, Low PNI}	0.007	0.344	0.019	2.714	11
14	{N3, High EBV, High LDH, Low ALC}	0.007	0.344	0.019	2.714	11
15	{N3, High EBV, Low WBC}	0.007	0.343	0.021	2.707	12
16	{N3, High LDH, Low ALC}	0.008	0.341	0.025	2.696	14
17	{N3, High EBV, Low ALC}	0.010	0.340	0.028	2.688	16
18	{N3, HBsAg (-), High LDH}	0.012	0.339	0.034	2.679	19
19	{N3, HBsAg (-), High ALC}	0.007	0.333	0.022	2.632	12
20	{N3, HBsAg (-), High WBC}	0.010	0.333	0.031	2.632	17
21	{N3, Low ALC, Low PNI}	0.010	0.333	0.029	2.632	16
22	{N3, High EBV, Low PNI}	0.009	0.326	0.028	2.574	15
23	{N1, HBsAg (+), High EBV, Low ALC}	0.008	0.325	0.024	2.566	13
24	{N3, High EBV, High LDH}	0.010	0.321	0.032	2.532	17
25	{N3, HBsAg (-), High PNI}	0.008	0.317	0.025	2.503	13
26	{N3, Low PNI}	0.011	0.316	0.035	2.493	18
27	{N3, High LDH}	0.013	0.314	0.042	2.481	22
28	{N1, HBsAg (+), High EBV, Low PNI}	0.007	0.314	0.021	2.481	11
29	{N3, High EBV}	0.015	0.312	0.047	2.461	24
30	{N3, Low ALC}	0.012	0.311	0.037	2.459	19

No.	LHS (Rules)	Support	Confidence	Coverage	Lift	Count
	Top 30 rules associated with DMFS					
	**Top 10 rules associated HBsAg positivity with DMFS**					
1	{HBsAg (+), Low ALC, High EBV}	0.012	0.308	0.039	2.429	20
2	{HBsAg (+), Low PNI, High EBV}	0.010	0.266	0.039	2.097	17
3	{HBsAg (+), High EBV}	0.016	0.248	0.064	1.955	26
4	{HBsAg (-), High PNI, High ALC, High EBV}	0.021	0.207	0.102	1.635	35
5	{High PNI, Low ALC, High EBV}	0.010	0.203	0.048	1.599	16
6	{High PNI, High EBV}	0.033	0.199	0.167	1.573	55
7	{HBsAg (-), High ALC, High EBV}	0.025	0.198	0.128	1.564	42
8	{Low ALC, High EBV}	0.039	0.196	0.198	1.545	64
9	{High EBV}	0.068	0.193	0.351	1.527	112
10	{HBsAg (+), Low PNI, Low ALC}	0.014	0.184	0.076	1.453	23

Note 1. The figures for the top 10 association rules are presented in **Figure 2A**.

Note 2. All rhs were defined as distant metastases.

Note 3. T and N stages were recorded according to the 8th edition AJCC/UICC staging system.

Note 4. The classification of inflammatory hematological indicators, including ALC, LDH, WBC, and PNI, were divided by the median value. High refers to those exceeding the median value, and low refers to those equal to or less than the median value. The plasma EBV DNA level classification was divided by 4×10^3 copies/mL. High EBV indicated plasma EBV DNA level > 4×10^3 copies/mL, and vice versa.

Note 5. All rules were completely repeatable by setting random seed = 2023 (the year this study began), using R's apriori {arules} package.

ALC = absolute lymphocyte count; EBV = pre-treatment plasma EBV DNA level; lhs = left-hand side; HBsAg = hepatitis B surface antigen; rhs = right-hand side; LDH = lactate dehydrogenase; PNI = prognostic nutritional index; WBC = white blood cell; + = positive; - = negative

SUPPLEMENTARY TABLE 2. Interaction analysis among HBsAg, EBV, and ALC

Variables	DMFS		Variables	DMFS		Variables	DMFS	
	HR (95% CI)	*P*		HR (95% CI)	*P*		HR (95% CI)	*P*
HBsAg (+) × Low ALC (HL)	2.67 (1.19, 5.99)	0.017	HBsAg (+) × High EBV (HE)	2.27 (1.08, 4.81)	0.031	Low ALC × High EBV	0.77 (0.44, 1.34)	0.357
HBsAg (-/+)	0.59 (0.29, 1.17)	0.130	HBsAg (-/+)	0.69 (0.38, 1.27)	0.231	ALC (≤ 1.9 vs. > 1.9)*	1.27 (0.84, 1.91)	0.257
ALC (≤ 1.9 vs. > 1.9)*	0.94 (0.69, 1.27)	0.681	EBV (≤ 4 vs. > 4)#	1.34 (0.97, 1.85)	0.072	EBV (≤ 4 vs.> 4)#	1.81 (1.17, 2.81)	0.008
EBV (≤ 4 vs.> 4)#	1.54 (1.15, 2.06)	0.004						
T stage			T stage			T stage		
T1	1 (reference)		T1	1 (reference)		T1	1 (reference)	
T2	1.62 (0.95, 2.78)	0.079	T2	1.63 (0.95, 2.8)	0.075	T2	1.58 (0.92, 2.71)	0.098
T3	1.61 (1.03, 2.5)	0.035	T3	1.62 (1.04, 2.52)	0.032	T3	1.61 (1.03, 2.5)	0.035
T4	2.59 (1.65, 4.06)	< 0.001	T4	2.67 (1.7, 4.18)	< 0.001	T4	2.58 (1.64, 4.04)	< 0.001
N stage			N stage			N stage		
N0	1 (reference)		N0	1 (reference)		N0	1 (reference)	
N1	2.03 (1.19, 3.46)	0.009	N1	2 (1.17, 3.41)	0.011	N1	2 (1.17,3.4)	0.011
N2	3.3 (1.85, 5.88)	< 0.001	N2	3.29 (1.85, 5.86)	< 0.001	N2	3.23 (1.81,5.77)	< 0.001
N3	5.25 (2.78, 9.93)	< 0.001	N3	5.14 (2.72, 9.7)	< 0.001	N3	5.07 (2.68,9.59)	< 0.001

Note 1. # EBV (×1000 copies/mL), * ALC ($\times10^9$/L).

Note 2. HR and *P* values were calculated using multivariate Cox regression analysis.

ALC = absolute lymphocyte count; CI = confidence interval; DMFS = distant metastasis-free survival; EBV = pre-treatment plasma EBV DNA level; HBsAg = hepatitis B surface antigen; HE = the interaction between HBsAg (+) and high EBV; HL = interaction between HBsAg (+) and low ALC; HR = hazard ratio; +, positive; -, negative

SUPPLEMENTARY TABLE 3. C-index of considering the item of HL and HE

DMFS prediction	Total cohort (n = 1650)	Training cohort (n = 825)	Testing cohort (n = 825)
**Proposed model**			
C-index	0.705	0.712	0.695
HR (95% CI)	(0.671–0.738)	(0.668–0.757)	(0.645–0.745)
**TN+EBV model**			
C-index	0.696	0.701	0.691
HR (95% CI)	(0.662–0.729)	(0.655–0.746)	(0.641–0.741)
P	0.762	0.25	0.361
**TN model**			
C-index	0.682	0.688	0.678
HR (95% CI)	(0.648–0.716)	(0.644–0.732)	(0.623–0.730)
P	0.006	0.002	0.047

Note 1. The proposed model was established incorporating HBsAg, HL, HE, ALC, EBV status, as well as tumor (T) and Node (N) stages. The TN+EBV model was established based on EBV status and T and N stages. The TN model was established considering the T and N stages.

Note 2. Training and testing cohorts were divided in a 1:1 ratio from the samples in the two hospitals.

Note 3. C-indices were compared pairwise using U-statistics computed using the rcorrp.cens function of the Hmisc package in R.

ALC = absolute lymphocyte count; DMFS = distant metastasis-free survival; EBV = pre-treatment plasma EBV DNA level; HBsAg = hepatitis B surface antigen; HE = interaction between HBsAg (+) and high EBV; HL = interaction between HBsAg (+) and low ALC

SUPPLEMENTARY TABLE 4. The incidence rate of adverse events occurring among patients with interaction effects when treated with or without induction chemotherapy

Adverse events	HL (+)		P	HE (+)		
	non_IC	IC		non_IC	IC	P
**Hematological**						
Neutropenia			< 0.001			< 0.001
None	24 (35.3%)	9 (13.6%)		14 (35.9%)	5 (8.3%)	
Grade 1/2	38 (55.9%)	21 (31.8%)		21 (53.8%)	21 (35%)	
Grade 3/4	6 (8.8%)	36 (54.5%)		4 (10.3%)	34 (56.7%)	
Febrile neutropenia			0.932			0.046
None	68 (100%)	66 (100%)		39 (100%)	60 (100%)	
Neutropenic infection			0.932			0.046
None	68 (100%)	66 (100%)		39 (100%)	60 (100%)	
Leucopenia			< 0.001			< 0.001
None	7 (10.3%)	1 (1.5%)		5 (12.8%)	1 (1.7%)	
Grade 1/2	47 (69.1%)	31 (47%)		26 (66.7%)	24 (40%)	
Grade 3/4	14 (20.6%)	34 (51.5%)		8 (20.5%)	35 (58.3%)	
Anemia			0.001			0.006
None	17 (25%)	2 (3%)		9 (23.1%)	2 (3.3%)	
Grade 1/2	49 (72.1%)	62 (93.9%)		27 (69.2%)	55 (91.7%)	
Grade 3/4	2 (2.9%)	2 (3%)		3 (7.7%)	3 (5%)	
Thrombocytopenia			0.223			0.293
None	46 (67.6%)	35 (53%)		25 (64.1%)	29 (48.3%)	
Grade 1/2	15 (22.1%)	22 (33.3%)		9 (23.1%)	19 (31.7%)	
Grade 3/4	7 (10.3%)	9 (13.6%)		5 (12.8%)	12 (20%)	
Lymphopenia			0.450			0.746
None	1 (1.5%)	1 (1.5%)		2 (5.1%)	1 (1.7%)	
Grade 1/2	16 (23.5%)	9 (13.6%)		8 (20.5%)	12 (20%)	
Grade 3/4	51 (75%)	56 (84.8%)		29 (74.4%)	47 (78.3%)	
**Non-hematological**						
Stomatitis (mucositis)			0.882			0.202
None	12 (17.6%)	13 (19.7%)		7 (17.9%)	9 (15%)	
Grade 1/2	38 (55.9%)	38 (57.6%)		20 (51.3%)	41 (68.3%)	
Grade 3/4	18 (26.5%)	15 (22.7%)		12 (30.8%)	10 (16.7%)	
Vomiting or nausea			0.006			< 0.001
None	30 (44.1%)	14 (21.2%)		22 (56.4%)	12 (20%)	
Grade 1/2	35 (51.5%)	51 (77.3%)		17 (43.6%)	48 (80%)	
Grade 3/4	3 (4.4%)	1 (1.5%)		0 (0%)	0 (0%)	
Dry mouth			0.055			0.301
None	30 (44.1%)	40 (60.6%)		17 (43.6%)	33 (55%)	
Grade 1/2	38 (55.9%)	26 (39.4%)		22 (56.4%)	27 (45%)	
Grade 3/4	0	0		0	0	

Adverse events	HL (+)		*P*	HE (+)		*P*
	non_IC	IC		non_IC	IC	
Diarrhea			0.033			0.044
None	66 (97.1%)	57 (86.4%)		39 (100%)	52 (86.7%)	
Grade 1/2	2 (2.9%)	6 (9.1%)		0 (0%)	7 (11.7%)	
Grade 3/4	0 (0%)	3 (4.5%)		0 (0%)	1 (1.7%)	
Skin			0.403			0.593
None	13 (19.1%)	17 (25.8%)		9 (23.1%)	15 (25%)	
Grade 1/2	55 (80.9%)	49 (74.2%)		29 (74.4%)	45 (75%)	
Grade 3/4	0 (0%)	0 (0%)		1 (2.6%)	0 (0%)	
Hair loss			0.025			0.384
None	63 (92.6%)	52 (78.8%)		31 (79.5%)	52 (86.7%)	
Grade 1/2	5 (7.4%)	14 (21.2%)		8 (20.5%)	7 (11.7%)	
Grade 3/4	0 (0%)	0 (0%)		0 (0%)	1 (1.7%)	
Fatigue			0.808			1
None	59 (86.8%)	56 (84.8%)		33 (84.6%)	50 (83.3%)	
Grade 1/2	9 (13.2%)	10 (15.2%)		6 (15.4%)	10 (16.7%)	
Grade 3/4	0 (0%)	0 (0%)		0 (0%)	0 (0%)	
Infection or fever			0.717			1
None	63 (92.6%)	63 (95.5%)		37 (94.9%)	56 (93.3%)	
Grade 1/2	5 (7.4%)	3 (4.5%)		2 (5.1%)	3 (5%)	
Grade 3/4	0 (0%)	0 (0%)		0 (0%)	1 (1.7%)	
Allergic reaction			0.272			1
None	65 (95.6%)	61 (92.4%)		36 (92.3%)	56 (93.3%)	
Grade 1/2	2 (2.9%)	5 (7.6%)		3 (7.7%)	4 (6.7%)	
Grade 3/4	1 (1.5%)	0 (0%)		0 (0%)	0 (0%)	
Deafness or otitis			0.163			1
None	53 (77.9%)	58 (87.9%)		34 (87.2%)	51 (85%)	
Grade 1/2	15 (22.1%)	8 (12.1%)		5 (12.8%)	8 (13.3%)	
Grade 3/4	0 (0%)	0 (0%)		0 (0%)	1 (1.7%)	
Esophagus discomfort			0.139			0.39
None	18 (26.5%)	24 (36.4%)		11 (28.2%)	25 (41.7%)	
Grade 1/2	49 (72.1%)	38 (57.6%)		26 (66.7%)	32 (53.3%)	
Grade 3/4	1 (1.5%)	4 (6.1%)		2 (5.1%)	3 (5%)	
Throat discomfort			0.666			1
None	53 (77.9%)	54 (81.8%)		32 (82.1%)	49 (81.7%)	
Grade 1/2	15 (22.1%)	12 (18.2%)		7 (17.9%)	11 (18.3%)	
Grade 3/4	0 (0%)	0 (0%)		0(0%)	0 (0%)	
Nephrotoxic event			0.111			1
None	67 (98.5%)	61 (92.4%)		37(94.9%)	56 (93.3%)	
Grade 1/2	1 (1.5%)	5 (7.6%)		2(5.1%)	4 (6.7%)	
Grade 3/4	0 (0%)	0 (0%)		0 (0%)	0 (0%)	

Adverse events	HL (+)		*P*	HE (+)		*P*
	non_IC	IC		non_IC	IC	
Hepatotoxic event			0.18			0.366
None	60 (88.2%)	50 (75.8%)		32 (82.1%)	45 (75%)	
Grade 1/2	5 (7.4%)	9 (13.6%)		6 (15.4%)	9 (15%)	
Grade 3/4	3 (4.4%)	7 (10.6%)		1 (2.6%)	6 (10%)	
Digestive discomfort			0.092			0.011
None	21 (30.9%)	12 (18.2%)		15 (38.5%)	8 (13.3%)	
Grade 1/2	43 (63.2%)	44 (66.7%)		23 (59%)	48 (80%)	
Grade 3/4	4 (5.9%)	10 (15.2%)		1 (2.6%)	4 (6.7%)	
Cardiac discomfort			1			1
None	67 (98.5%)	65 (98.5%)		39 (100%)	59 (98.3%)	
Grade 1/2	1 (1.5%)	1 (1.5%)		0 (0%)	1 (1.7%)	
Grade 3/4	0 (0%)	0 (0%)		0 (0%)	0 (0%)	

* p values were calculated for acute adverse event distribution between patients with nasopharyngeal carcinoma treated with and without induction chemotherapy in hospital 1, using Fisher's exact test or the chi-squared test for categorical variables.

The acute adverse events were graded according to the Common Terminology Criteria for Adverse Events (CTCAE) Version 4.0.

HE = interaction effect of HBsAg and high EBV; HL = interaction effect of HBsAg and low ALC; IC = induction chemotherapy
